# Metabolic Response of Black Tiger Shrimp (*Penaeus monodon*) to Acute Ammonia Nitrogen Stress

**DOI:** 10.3390/biology14050501

**Published:** 2025-05-04

**Authors:** Yangyang Ding, Shigui Jiang, Song Jiang, Yundong Li, Qibin Yang, Lishi Yang, Jianhua Huang, Jianzhi Shi, Pengying Li, Hongshan Diao, Falin Zhou

**Affiliations:** 1 Key Laboratory of South China Sea Fishery Resources Exploitation and Utilization, Ministry of Agriculture, South China Sea Fisheries Research Institute, Chinese Academy of Fishery Sciences, Guangzhou 510300, China; dingyangyang93@163.com (Y.D.); jiangsg@21cn.com (S.J.); tojiangsong@163.com (S.J.); liyd2019@163.com (Y.L.); yangqibin1208@163.com (Q.Y.); yangls2016@163.com (L.Y.); shijianzhi1989@163.com (J.S.); lipengyingcafs@163.com (P.L.); 17875808801@163.com (H.D.); 2 State Key Laboratory of Mariculture Biobreeding and Sustainable Goods, Yellow Sea Fisheries Research Institute, Chinese Academy of Fishery Sciences, Qingdao 266071, China; 3 Shenzhen Base of South China Sea Fisheries Research Institute, Chinese Academy of Fishery Sciences, Shenzhen 518121, China; huangjianhua@scsfri.ac.cn

**Keywords:** black tiger shrimp (*Penaeus monodon*), ammonia nitrogen stress, metabolic response, antioxidant enzymes, caspase

## Abstract

High concentrations of ammonia nitrogen could result in the death of aquatic animals and cause a huge economic loss in the aquaculture industry. However, the metabolic responses to acute ammonia nitrogen stress remain largely unknown in *Penaeus monodon*. In this study, we first investigated the histological change in tissues in *Penaeus monodon* under 96 h acute ammonia nitrogen stress. The result of the paraffin section showed that acute ammonia nitrogen stress induced severe epithelial detachment and lumen dilatation of the hepatopancreas, swollen and hemocyte infiltration of the gills, and mucosa exfoliation and shortened villi of the intestine in *Penaeus monodon*, suggesting the impairment of the normal physiological function in these tissues. We next examined the change in the metabolic product in the plasma and the enzyme activity in the hepatopancreas after ammonia nitrogen stress. Upon ammonia stress, both the concentration of ammonia and urea nitrogen significantly increased, while there was no significant increase in the concentration of uric acid, which is consistent with the results that the enzyme activity of glutamine synthetase (GS), glutamate dehydrogenase (GDH), and aspartate transaminase (GOT) became significantly elevated and the enzyme activity of adenosine deaminase (ADA) in the purine metabolism pathway significantly decreased after ammonia stress, suggesting that shrimp could convert excessive ammonia to urea for ammonia detoxification through the ammonia–nitrogen metabolism pathways. Interestingly, we also observed a significant increase in superoxide dismutase (SOD) activity, suggesting a potential role of this antioxidant enzyme in the clearance of reactive oxygen species (ROS) induced via ammonia stress. Moreover, we found that acute ammonia nitrogen stress inhibited the enzyme activity of caspase 3 and caspase 8, suggesting an important role of apoptosis in protecting *Penaeus monodon* against acute ammonia stress. Overall, our findings revealed that *Penaeus monodon* may employ metabolic and purine pathways and undergo oxidative stress and apoptosis for ammonia detoxification under ammonia nitrogen stress, thus providing new insight into the metabolic response of shrimp to acute ammonia stress.

## Simple Summary

Ammonia nitrogen is an extremely harmful pollutant in the aquatic environments, which could cause the death of the shrimp and thus hinder the promotion of new varieties of shrimp, resulting in a huge economic loss. However, the metabolic response of black tiger shrimp (*Penaeus monodon*) to acute ammonia nitrogen stress remains unclear. In this study, the histological change in the hepatopancreas, gills, and intestine, the metabolic product of ammonia nitrogen in the plasma, and the enzyme activity in the hepatopancreas were investigated. The results showed that acute ammonia nitrogen stress could result in severe tissue damage in black tiger shrimp. Meanwhile, black tiger shrimp could convert excessive ammonia nitrogen to urea for detoxification. In addition, black tiger shrimp might modulate antioxidants and apoptosis to reduce the damage caused by ammonia nitrogen.

## 1. Introduction

In aquatic environments, ammonia nitrogen (ammonia-N) is a common toxic and harmful substance, and its concentration is an important indicator of the water quality [1]. The origin of ammonia-N is mostly from the natural decomposition of organic matter, the metabolic byproducts of protein catabolism yielding, and the excessive effluent discharge [2]. Ammonia nitrogen refers to the total ammonia in the water, which primarily exists in two forms: ammonium (NH_4_^+^) and molecular ammonia (NH_3_). These two forms are in a state of dynamic equilibrium, and the NH_4_^+^ to NH_3_ ratios are affected by the salinity, temperature, and especially pH, which are also the determinants of ammonia-N toxicity [3]. An inverse relationship exists between salinity and ammonia-N toxicity; that is, a lower salinity increases the NH_4_^+^ to NH_3_ ratio and thus makes it more toxic [4]. When the temperature increased, the ratio of NH_4_^+^ to NH_3_ decreased so the ammonia-N became more toxic [2]. A higher pH will increase the ammonia-N toxicity due to the NH_3_ form becoming more prevalent than NH_4_^+^ [5]. NH_3_ is uncharged and highly lipophilic, allowing it to penetrate cell membranes and exert toxic effects on organisms, thus being more toxic than NH_4_^+^ [6]. Chronic ammonia-N toxicity can cause tissue damage, enhance gill permeability, impair ion exchange functions, and reduce the growth rate and reproductive capacity of crustaceans. It can also increase their susceptibility to pathogens [7]. Acute ammonia-N toxicity can cause aquatic organisms to experience convulsions, persistent hyperexcitability, loss of balance in water, hypoxia, and death. Additionally, it can inhibit the activity of various enzymes, cause severe oxidative damage, and trigger apoptosis [8,9].

In crustaceans, ammonia nitrogen is primarily generated through the transamination and deamination of amino acids via specific enzymes, and these reactions are usually reversible [10]. For example, glutamine synthetase (GS) can utilize NH_4_^+^ to synthesize glutamine, and glutamate dehydrogenase (GDH) can use NH_4_^+^ to synthesize glutamate. Thus, crustaceans detoxify ammonia nitrogen by synthesizing non-toxic amino acids such as glutamine and glutamate [11]. GS is an important enzyme in ammonia nitrogen metabolism in crustaceans and is widely found in plants and animals [12]. GDH is a mitochondrial enzyme widely present in plants, animals, and microorganisms. It is a key enzyme in amino acid catabolism, distributed in tissues such as the liver, kidney, and brain in animals, where it exhibits a high activity [13]. GDH is the only enzyme capable of using both NAD⁺ and NADP⁺ as redox cofactors [14]. Studies have shown that when GDH uses NADP⁺ as a cofactor, it primarily catalyzes glutamate synthesis, whereas with NAD⁺, it catalyzes the oxidative deamination of glutamate [15].

Under normal conditions, the main metabolite of amino acids in crustaceans is ammonia nitrogen, with only a small amount as urea and uric acid [16]. However, studies have shown that when the external environment (e.g., salinity, pH, or ammonia nitrogen) changed, urea and nitrite excretion significantly increased in shrimp [17]. Aspartate transaminase (GOT/AST) can catalyze the reaction between glutamate and oxaloacetate to produce aspartate, which contributes to ammonia production through transamination [16]. In the mammalian mitochondria, aspartate serves as a direct source of amino groups during urea formation [18]. Thus, GOT is closely related to ammonia production and excretion. Xanthine oxidoreductase (XOD) is a member of the molybdenum-containing enzyme family and is widely distributed in the various tissues of animals. It primarily participates in the physiological processes of hypoxanthine metabolism to xanthine and xanthine oxidation to uric acid in mammals [19]. XOD exists in two main forms: xanthine dehydrogenase (XDH) and xanthine oxidase (XO) [20]. These enzymes are the key components in the purine metabolism pathway, where uric acid is the final metabolic product, and reactive oxygen species (ROS) are metabolic byproducts [21]. Adenosine deaminase (ADA) can catalyze the irreversible deamination of adenosine to inosine, and the final metabolite is uric acid [22].

Ammonia stress can also increase the concentration of reactive oxygen species (ROS) in the organism and thus lead to oxidative stress in organisms [23]. The excessive production of ROS can lead to the severe impairment of cellular function. Therefore, the organism has evolved antioxidant systems to maintain the intracellular redox homeostasis as well as to maintain the normal cellular function [24]. Among them, superoxide dismutase (SOD) can catalyze the conversion of harmful superoxide radicals and oxygen radicals into less toxic hydrogen peroxide (H_2_O_2_), while catalase (CAT) can catalyze the conversion of toxic H_2_O_2_ into nontoxic water, and they play important roles in the protection of living organisms as important components of biological antioxidant systems [19]. In addition, programmed cell death plays important roles in embryonic development, organ maintenance, aging, and the coordination of immune responses and autoimmunity, and apoptosis is one of the most well-understood types [25]. Cells undergoing apoptosis not only encourage phagocytosis by phagocytes but also actively reduce inflammation and aid in the tissue repair within the surrounding area [25].

Black tiger shrimp (*Penaeus monodon*) is the largest species within the genus Penaeus, primarily distributed along the Indo-West Pacific coasts. As one of the three major cultured shrimp species globally, black tiger shrimp is a key marine aquaculture species in the South China Sea and Southeast Asia, holding significant economic value. In culture conditions, due to the intensification and high-density farming of black tiger shrimp, ammonia nitrogen has become one of the primary pollutants in the aquaculture water environment, which can directly damage the immune system of black tiger shrimp, indirectly increasing its susceptibility to pathogens. Acute ammonia nitrogen stress can induce oxidative stress, apoptosis, and purine metabolism detoxification pathways in black tiger shrimp [9]. However, the metabolic response of black tiger shrimp to acute ammonia nitrogen stress remains largely unknown. We hypothesized that black tiger shrimp can convert excessive ammonia to harmless substances for detoxification through the ammonia-metabolism pathway and purine-metabolism pathway. Meanwhile, shrimp can also modulate the antioxidants and apoptosis to reduce the damage caused by ammonia nitrogen. In this study, we investigated the products of ammonia nitrogen metabolism, the activity of enzymes related to ammonia nitrogen and the purine-metabolism pathway, as well as antioxidant and apoptosis-related enzymes after ammonia nitrogen stress, which will enhance the understanding of the metabolism response of black tiger shrimp to ammonia stress and facilitate the process of breeding new strains of ammonia-tolerant shrimp.

## 2. Materials and Methods

### 2.1. Experimental Animals

Healthy juvenile black tiger shrimp were obtained from the experimental base of the South China Sea Fisheries Research Institute (Shenzhen, China). Shrimp (average body length: 11 ± 0.5 cm, body weight: 21 ± 1 g) were selected and acclimatized for 2 weeks in black tanks containing 500 L of filtered seawater (30 ± 1‰, temperature 28 ± 1 °C, pH 7.6 ± 0.3, and dissolved oxygen ≥ 6.8 mg/L). Shrimp were fed daily with commercial pellets (Dongteng, Guangzhou, China) at a rate of 1% body weight per day. Half of the seawater was replaced daily to maintain the water quality. Shrimp were anesthetized with an overdose of eugenol before being sacrificed. All efforts were made to minimize the suffering of the animals.

### 2.2. 96 h Acute Ammonia Nitrogen Stress

The 96 h median lethal concentration (LC_50_) of ammonia nitrogen for black tiger shrimp was determined through a preliminary experiment according to previous studies, with minor modification [26]. The LC_50_ was calculated using linear interpolation and determined to be 65.0 mg/L ammonium chloride (NH_4_Cl) (Pubo, Guangzhou, China) (Figure S1). In the acute ammonia nitrogen stress experiment, shrimp were challenged with 65.0 mg/L ammonium chloride, with three replicates (50 shrimp per replicate). Shrimp without ammonium chloride treatment were set as the control group, with three replicates (50 shrimp per replicate). Samples were collected at 0, 6, 12, 24, 48, 72, and 96 h after ammonia nitrogen stress. Three shrimp were randomly selected from each replicate for mixed sampling, and a total of nine shrimp were used for analysis at each time point. The hemolymph was drawn using a 0.2 mL disposable syringe pre-washed with anticoagulant. Then, the hemolymph samples were centrifuged at 3000× rpm for 10 min at 4 °C to separate the plasma. The plasma samples were stored at −80 °C before their analysis. Mixed samples of hepatopancreas tissue were collected from three shrimp per time point and stored in liquid nitrogen immediately before enzyme activity analysis.

### 2.3. Histology Analysis

The hepatopancreas, gill, and intestine tissues of black tiger shrimp from the control group and the ammonia nitrogen stress group (96 h after stress) were dissected and fixed in 4% neutral buffered formalin overnight at 4 °C for the preparation of paraffin sections. To study the effect of ammonia stress on the tissue of black tiger shrimp, the embedded sections were stained with hematoxylin and eosin (HE) to visualize the morphological structure. Images were acquired at a 200× magnification and analyzed using the Nikon DS-U3 imaging system. The histopathological severity score was developed based on previous studies in the liver, gills, and intestine, with minor modification [27,28,29]. The following parameters were scored individually and added to generate the final pathology score: separation of the basement membrane from epithelial cells, lumen dilatation, filaments’ level of swelling, hemocyte infiltration, mucosa exfoliation, shortened villi, and the percentage involvement of the tissue. The scoring method is presented in Table S1.

### 2.4. Measurement of Ammonia, Urea, and Uric Acid in the Plasma

Plasma samples from each time point were used to measure the metabolic products of black tiger shrimp under ammonia nitrogen stress. The Nanjing Jiancheng Bioengineering Institute (Nanjing, China) kits (Blood Ammonia assay kit (A086-1-1), Urea Assay Kit (C013-2-1), and Uric acid (UA) Test Kit (C012-2-1)) were employed to detect the concentration of ammonia, urea, and uric acid in the plasma samples, respectively, following the manufacturer’s instructions. The concentration of ammonia in the plasma was measured through the Berthelot reaction. Ammonium was oxidized to chloramine and phenol, and then they were catalyzed to form blue indophenol [30]. The concentration of urea in the plasma can be hydrolyzed by urease to produce ammonium and carbon dioxide, and then, ammonium can be detected [31]. The uric acid in the plasma was oxidized to allantoin and hydrogen peroxide, and then the hydrogen peroxide can be detected [32].

### 2.5. Measurement of the Enzyme Activity in the Hepatopancreas

Hepatopancreas samples from each time point were used to measure the activity of ammonia nitrogen metabolism-related enzymes (including GS, GDH, GOT/AST, ADA, and XOD), the antioxidant enzymes, superoxide dismutase and catalase (SOD and CAT), and the apoptosis-related enzymes (caspase 3 and caspase 8). The same weight of hepatopancreas tissues was used to detect the protein concentration of each sample by using the Bradford method. Briefly, the hepatopancreas was homogenized before centrifugation, and then, the supernatant was mixed with Coomassie Brilliant Blue to detect the absorbance. The Nanjing Jiancheng Bioengineering Institute kits (glutamine synthetase assay kit; glutamate dehydrogenase assay kit; aspartate aminotransferase assay kit; xanthine oxidase assay kit; adenosine deaminase assay kit; superoxide dismutase assay kit; catalase assay kit; caspase 3 activity assay kit; caspase 8 activity assay kit) were employed to detect the enzymes’ activity, respectively, according to the manufacturer’s instructions.

### 2.6. Statistical Analysis

An independent *t* test was used to analyze the differences in the histopathological severity score between the groups. The data of the metabolic products and the enzyme activities were analyzed using one-way ANOVA followed by Tukey’s multiple comparison test in SPSS 23.0 and presented as the mean ± the standard error of the mean (mean ± SEM). Figures were made using GraphPad Prism 8 software. It was considered statistically significant when *p* < 0.05.

## 3. Results

### 3.1. Effects of Ammonia Nitrogen Stress on Shrimp Tissues

**Figure 1:**
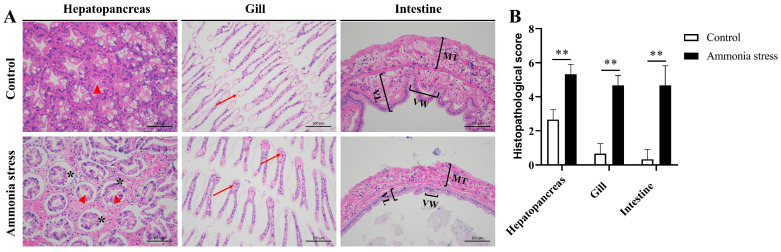
Histological changes in the hepatopancreas, gills, and intestine of black tiger shrimp under acute ammonia stress. (**A**) Images of hematoxylin and eosin staining on black tiger shrimp hepatopancreas (**left**), gill (**middle**), and intestine (**right**) paraffin sections from the control shrimp (**upper**) and ammonia nitrogen stress shrimp (**lower**) (*n* = 3 per group). Black asterisk indicates severe separation of the basement membrane from epithelial cells. The red triangle indicates lumen dilatation. Red arrows indicate infiltrated hemocytes in the gills. Mucosal thickness (MT), villi length (VL), and villi width (VW). Magnification, 200×. Scale bar, 100 μm. (**B**) Histopathological severity score of hepatopancreas, gills, and intestine in control shrimp and shrimp after 96 h of acute ammonia nitrogen stress (*n* = 3 per group). ** *p* < 0.01.

To study the effect of ammonia stress on the tissues of black tiger shrimp, the paraffin sections of hepatopancreas, gill, and intestine tissues from the control group and the ammonia nitrogen stress group (96 h after stress) were stained with hematoxylin and eosin and analyzed. In the hepatopancreas, we observed the severe separation of the basement membrane from epithelial cells and lumen dilatation after acute ammonia nitrogen stress (Figure 1A, left). In the gills, the gill filaments were swollen and hemocytes infiltrated the gill tissue after ammonia nitrogen stress (Figure 1A, middle). In the intestine, mucosa showed exfoliation from the epithelial cells, and the villi became shortened after ammonia nitrogen stress (Figure 1A, right). We observed significant changes in the histopathological severity score in the hepatopancreas, gills, and intestine from the control shrimp and shrimp after 96 h of acute ammonia nitrogen stress (Figure 1B). These results indicated that ammonia nitrogen stress induced severe histological damage in the black tiger shrimp.

### 3.2. Effects of Ammonia Nitrogen Stress on the Metabolic Product in the Plasma

**Figure 2:**
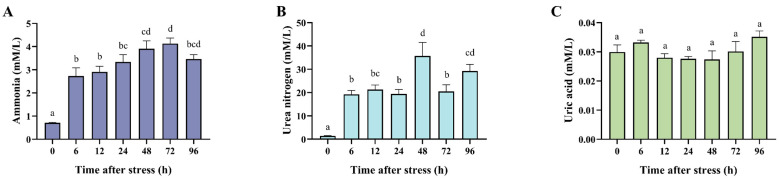
The metabolic products in the hemolymph of black tiger shrimp after 96 h of acute ammonia nitrogen stress. (**A**) The histogram represents the concentration of ammonia in the plasma of black tiger shrimp. (**B**) The histogram represents the concentration of urea nitrogen in the plasma of black tiger shrimp. (**C**) The histogram represents the concentration of uric acid in the plasma of black tiger shrimp. Each bar represents the mean ± standard error of the mean (mean ± SEM) (*n* = 9). Data with different letters are significantly different among groups (*p* < 0.05).

In order to study the metabolic responses of shrimp to ammonia stress, we detected the concentration of ammonia, urea nitrogen, and uric acid in the plasma of black tiger shrimp after acute ammonia nitrogen stress. The results showed that both the concentration of ammonia and urea nitrogen significantly increased in the plasma after ammonia stress, while the levels of uric acid did not change (Figure 2). In detail, under acute ammonia nitrogen stress, the concentration of ammonia in the plasma rose rapidly, with a significant increase at 6 h (*p* < 0.05). The concentration of ammonia continued to rise, peaking at 48 h (4.13 mmol/L), which was 3.58 times higher than the 0 h control (Figure 2A). Although it declined afterward, the concentration at 96 h remained significantly higher than the control (*p* < 0.05). The concentration of urea nitrogen in the plasma followed a trend similar to ammonia (Figure 2B), with a significant increase after stress. The concentration at 6 h was significantly higher than the control (*p* < 0.05) and peaked at 48 h (39 mmol/L), which was 24.88 times higher than the control level. It subsequently decreased but remained significantly higher than the control at 96 h (*p* < 0.05). In contrast, the uric acid concentration in the plasma showed no significant differences at all the examined time points after ammonia stress (Figure 2C). These results indicated that black tiger shrimp could induce the conversion of excessive ammonia to urea rather than uric acid for ammonia detoxification under acute ammonia nitrogen stress.

### 3.3. Effect of Ammonia Nitrogen Stress on the Activity of Metabolic-Related Enzymes

**Figure 3:**
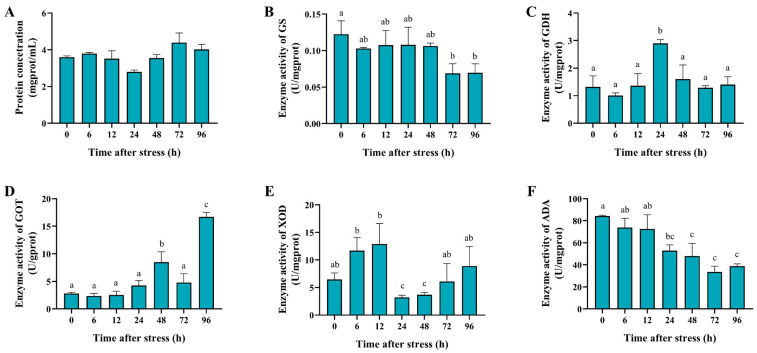
The enzyme activity of the ammonia nitrogen and purine metabolism-related enzymes in the hepatopancreas of black tiger shrimp after 96 h of acute ammonia nitrogen stress. (**A**) The histogram represents the protein concentration in the hepatopancreas of black tiger shrimp. (**B**) The histogram represents the enzyme activity of GS in the hepatopancreas of black tiger shrimp. (**C**) The histogram represents the enzyme activity of GDH in the hepatopancreas of black tiger shrimp. (**D**) The histogram represents the enzyme activity of GOT in the hepatopancreas of black tiger shrimp. (**E**) The histogram represents the enzyme activity of XOD in the hepatopancreas of black tiger shrimp. (**F**) The histogram represents the enzyme activity of ADA in the hepatopancreas of black tiger shrimp. Each bar represents the mean ± standard error of the mean (mean ± SEM) (*n* = 9). Data with different letters are significantly different among groups (*p* < 0.05).

To further explore the function of ammonia nitrogen and purine metabolism-related enzymes in the metabolic response to acute ammonia stress, we conducted enzyme activity analysis to reveal the change in the activity of GS, GDH, GOT/AST, XOD, and ADA after ammonia stress. The protein concentration of the hepatopancreas after ammonia nitrogen stress is shown in Figure 3A. The activities of ammonia-related metabolic enzymes exhibited the following patterns: The activity of glutamine synthetase (GS) significantly decreased at 96 h (*p* < 0.05) (Figure 3B). Glutamate dehydrogenase (GDH) activity was significantly elevated at 24 h (*p* < 0.01), with no significant difference observed at 96 h (Figure 3C). The activity of aspartate aminotransferase (GOT/AST) significantly increased (*p* < 0.01) (Figure 3D). The xanthine oxidase (XOD) activity was significantly higher than the control at 12 h (*p* < 0.01), then decreased, showing no significant difference from the control at 96 h (Figure 3E). The adenosine deaminase (ADA) activity was markedly reduced at 96 h (*p* < 0.01), showing a significant difference compared to the 0 h control (Figure 3F). The results of the increased activity of GOT supported the hypothesis that shrimp could convert ammonia to urea for detoxification. The change in GS and GDH activity also indicated that shrimp might convert ammonia to glutamine and glutamate for detoxification, warranting further research into their detoxification roles.

### 3.4. Effect of Ammonia Nitrogen Stress on the Activity of Antioxidant and Apoptosis-Related Enzymes

**Figure 4:**
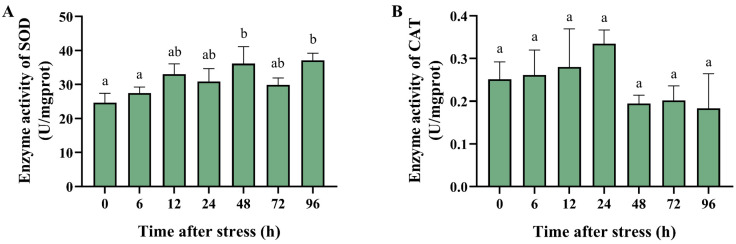
The enzyme activity of the antioxidant-related enzymes in the hepatopancreas of black tiger shrimp after 96 h of acute ammonia nitrogen stress. (**A**) The histogram represents the enzyme activity of SOD in the hepatopancreas of black tiger shrimp. (**B**) The histogram represents the enzyme activity of CAT in the hepatopancreas of black tiger shrimp. Each bar represents the mean ± standard error of the mean (mean ± SEM) (*n* = 9). Data with different letters are significantly different among groups (*p* < 0.05).

To reveal whether the antioxidant enzyme plays a role in the metabolic response to acute ammonia nitrogen stress, we further conducted the enzyme activity analysis of SOD and CAT in the hepatopancreas of black tiger shrimp after ammonia stress. The activities of the antioxidant enzymes, superoxide dismutase (SOD) and catalase (CAT), showed the following changes: SOD activity significantly increased at 48 and 96 h (*p* < 0.01) (Figure 4A). The CAT activity increased at 24 h but was not significantly different compared with that of the control group (Figure 4B). The results indicated that shrimp could activate SOD to prevent cellular oxidative damage due to ROS induced by high concentrations of ammonia nitrogen.

**Figure 5:**
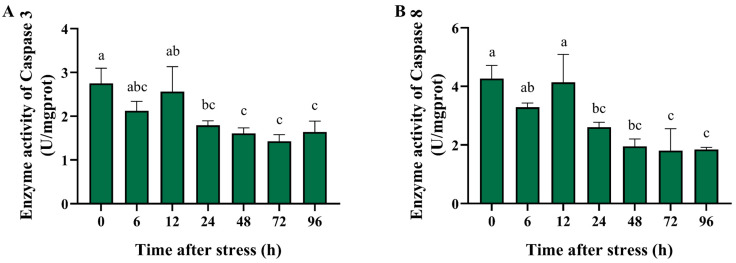
The enzyme activity of the apoptosis-related enzymes in the hepatopancreas of black tiger shrimp after 96 h of acute ammonia nitrogen stress. (**A**) The histogram represents the enzyme activity of caspase 3 in the hepatopancreas of black tiger shrimp. (**B**) The histogram represents the enzyme activity of caspase 8 in the hepatopancreas of black tiger shrimp. Each bar represents the mean ± standard error of the mean (mean ± SEM) (*n* = 9). Data with different letters are significantly different among groups (*p* < 0.05).

Previous studies found that ammonia nitrogen stress induced apoptosis in crustaceans [33]. We thus investigated the change in activity of the apoptosis-related enzymes caspase 3 and caspase 8 in the hepatopancreas of black tiger shrimp after ammonia stress. After high-concentration ammonia nitrogen stress, the activities of the apoptosis-related enzymes, caspase 3 and caspase 8, exhibited similar trends (Figure 5). The enzyme activities decreased immediately after the stress onset, increased at 12 h but not significantly differently from the control, and then showed a significant decline. At 96 h, their activities were significantly lower than those of the control (*p* < 0.01). The results indicated that ammonia stress suppressed the enzyme activity of the caspase family, and shrimp might modulate caspase-dependent apoptosis to reduce the damage caused by high concentrations of ammonia nitrogen.

## 4. Discussion

In crustaceans, 60–70% of nitrogen (N) is excreted as ammonia, with smaller amounts excreted as urea and uric acid [16]. To investigate the detoxification mechanisms of black tiger shrimp, this study measured the metabolic product and the metabolic-related enzyme activity following high-concentration ammonia stress. The results showed that the concentrations of ammonia and urea nitrogen in the hemolymph significantly increased, while the uric acid levels showed no significant changes, suggesting that black tiger shrimp might convert excess ammonia into urea for detoxification, possibly through the urea–ornithine pathway. In the swimming crab (*Portunus trituberculatus*), significant increases in ammonia and urea were also observed in the hemolymph under ammonia nitrogen stress [11]. Importantly, the key enzymes in the ammonia nitrogen metabolic pathway, such as GS, GDH, and GOT/AST, play critical roles in ammonia detoxification in crustaceans [34,35,36]. In this study, the enzyme activity of GDH and GOT significantly increased, while the GS activity significantly decreased under ammonia stress. GDH can facilitate the conversion of NH₄⁺ into non-toxic glutamate, while GOT transfers an amino group from glutamate to oxaloacetate, forming aspartate, a direct amino donor for urea synthesis [37]. The increased enzyme activity of GOT is in accordance with the observed increase in urea levels, indicating that black tiger shrimp may conduct ammonia detoxification through amino acid metabolic pathways. The change in GS and GDH activity suggested that black tiger shrimp may convert the excessive ammonia to glutamine and glutamate. Xanthine oxidase (XOD), a key enzyme in the purine metabolism pathway, catalyzes the conversion of hypoxanthine to xanthine and subsequently to uric acid [38]. In the land crab (*Gecarcoidea natalis*), excessive ammonia is converted to uric acid for detoxification [14]. Though the uric acid levels did not significantly change, the XOD activity significantly increased at 12 h after stress, suggesting a possible role for XOD in ammonia detoxification via purine metabolism. Conversely, ADA activity showed a significant decrease in this study. ADA can catalyze the conversion of adenosine into inosine; then, the inosine can produce hypoxanthine, which leads to the production of uric acid [39,40]. The results suggested that the unchanged levels of uric acid in the plasma after ammonia stress may be due to the suppression of the ADA activity of black tiger shrimp. In *Procambarus clarkii*, the ADA activity was significantly down-regulated to lower the purine metabolism after ammonia stress [41]. Moreover, uricase can oxidize uric acid, converting it into allantoin. Allantoin is subsequently hydrolyzed by allantoinase to form allantoic acid, which is ultimately converted into ammonia [42]. The suppression of ADA activity may also inhibit the excessive ammonia production in the body of black tiger shrimp.

The hepatopancreas is the main digestive and metabolic organ in shrimp, and thus plays a major role in the ammonia nitrogen stress response [43]. The gills and intestine are the external and internal borders of shrimp, which are in direct contact with the environment and thus the main target tissues of ammonia nitrogen [44]. In this study, acute ammonia nitrogen stress induced severe histopathological damage in the hepatopancreas, gills and intestine in black tiger shrimp, suggesting the impairment of the normal physiological function in these tissues. In *Litopenaeus vannamei*, ammonia exposure induced histopathological damage to the hepatopancreas and gills [45]. In addition, ammonia nitrogen stress could also induce tissue damage of the intestine mucosa and suppress the immune function of the intestine [46,47].

Ammonia nitrogen stress induces oxidative stress through increased reactive oxygen species (ROS) production [23]. For hosts, SOD and CAT play crucial roles in mitigating oxidative damage by converting ROS into less harmful molecules [24,48]. Thus, the activity of SOD and CAT is measured to indicate the cellular oxidant/antioxidant balance since they play important roles when protecting against cellular free radical damage [49]. The results showed that the enzyme activity of SOD significantly increased, suggesting that SOD may protect black tiger shrimp against oxidative damage caused by ammonia nitrogen stress through the clearance of ROS. Conversely, in *Litopenaeus vannamei* and *Marsupenaeus japonicus*, the activity of SOD and CAT was significantly decreased after ammonia exposure, while the malondialdehyde (MDA) levels significantly increased [45,50]. In *Ctenopharyngodon idellus*, the activity of SOD and CAT also significantly decreased when exposed to high environmental ammonia [51]. The increase in the activity of SOD but not CAT indicated that the oxidative stress produced by ROS was partially eliminated, but there may be residual and increased MDA levels. Interestingly, in *Takifugu obscurus*, the activity of SOD and CAT was significantly elevated after ammonia nitrogen stress [52]. So, we speculated that ammonia stress could induce oxidative stress in aquatic animals and influence the enzyme activity of antioxidants, but the responses of different animals may differ due to their different tolerance to ammonia and the different ways of treatment of ammonia in various studies.

Apoptosis plays a critical role in maintaining the homeostasis and defending against external and internal injuries in multicellular organisms. Apoptosis can be broadly categorized into caspase-independent apoptosis and caspase-dependent apoptosis [25]. Alterations in the activity of the caspase family of enzymes can be used as a marker for the initiation of apoptosis programs [52]. A recent study showed that the expression of caspase 2, which is involved in the initiation of apoptosis, and caspase 7, which is downstream of the apoptotic cascade, were significantly down-regulated in the black tiger shrimp under ammonia nitrogen stress [9]. Among the caspase family members, caspase 8 and caspase 3 are the most important initiator and executioner, respectively [25]. Therefore, in this study, we investigated the activity changes in caspase 8 and caspase 3 after ammonia nitrogen stress. The results showed that after a high concentration of ammonia nitrogen stress, the activities of both caspase 8 and caspase 3 significantly decreased. In *Litopenaeus vannamei*, ammonia exposure induces apoptosis in the hepatopancreas [53]. In *Ctenopharyngodon idellus*, the c-Myc-Bax-Caspase 9 apoptosis pathway was activated when exposed to high levels of ammonia [51]. The results suggested that ammonia stress suppressed the enzyme activity of the caspase family, and black tiger shrimp might modulate apoptosis to protect themselves under ammonia nitrogen stress.

## 5. Conclusions

In summary, acute ammonia nitrogen stress resulted in significant increases in ammonia and urea in the hemolymph, while uric acid remained unchanged in black tiger shrimp, suggesting that shrimp might convert excess ammonia into urea for ammonia detoxification. Importantly, the key metabolic and purine pathway enzymes, including GS, GDH, and GOT, showed notable activity changes, suggesting the vital roles of these enzymes in the metabolism of excessive ammonia. Moreover, the antioxidant enzyme SOD activity was significantly upregulated and the activities of the apoptosis-related proteins, caspase 8 and caspase 3, were significantly altered, indicating the responses involving anti-oxidative reaction and apoptosis. Overall, our findings revealed that black tiger shrimp undergo oxidative stress and apoptosis and employ metabolic and purine pathways for ammonia detoxification under ammonia nitrogen stress, thus providing new insights into the metabolic response of shrimp to acute ammonia stress.

## Data Availability

Data will be made available on request.

## Supplementary Materials

The following supporting information can be downloaded at: https://www.mdpi.com/article/10.3390/biology14050501/s1, Figure S1: The regression equation of the mortality and the concentration of NH_4_Cl in the 96 h acute ammonia stress experiment; Table S1: Histopathological severity score.
