# Supplementary material for: Metabolic Response of Black Tiger Shrimp (Penaeus monodon) to Acute Ammonia Nitrogen Stress
Source: Biology (Basel). 2025 May 4;14(5):501. doi: 10.3390/biology14050501 (PMC12109173; doi:10.3390/biology14050501)
Supplement: Supplementary file 1 [file biology-14-00501-s001.zip › biology-3578135-supplementary.pdf]

Table S1. Histopathological severity score

Tissue	Feature	Description	Score
**Hepatopancreas**	Separation of basement membrane from epithelial cells	Absence	0
		Presence	1
	Lumen dilatation	Absence	0
		Presence	1
	Percent involvement	0%	0
		1-25%	1
		26-50%	2
		51-75%	3
		76-100%	4
**Gill**	Filaments swollen	Absence	0
		Presence	1
	Hemocytes infiltration	Absence	0
		Presence	1
	Percent involvement	0%	0
		1-25%	1
		26-50%	2
		51-75%	3
		76-100%	4
**Intestine**	Mucosa exfoliation	Absence	0
		Presence	1
	Shortened villi	Absence	0
		Presence	1
	Percent involvement	0%	0
		1-25%	1
		26-50%	2
		51-75%	3
		76-100%	4

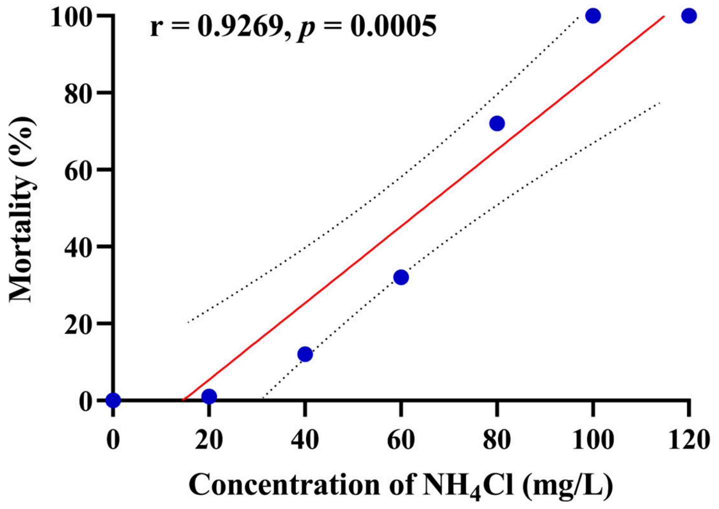

Figure S1. The regression equation of the mortality and the concentration of NH_4Cl in the 96 h acute ammonia stress experiment. To determine the LC_{50} in the 96 h acute ammonia stress experiment, seven ammonia nitrogen concentrations were used in the preliminary test. Each concentration contains 30 shrimp and a total of 210 shrimp were used. The regression equation of a straight line is y = 0.9964 x - 14.50 and the 95% confidence intervals of the slope is from 0.675 to 1.318.
